# The effect of physical exercise on apathy in older adults: a systematic review and meta-analysis

**DOI:** 10.3389/fpubh.2025.1617272

**Published:** 2025-08-21

**Authors:** Haichang Jia, Zhengyang Mei, Yang Luo, Xintong Mou, Jing Huang

**Affiliations:** College of Physical Education, Southwest University, Chongqing, China

**Keywords:** physical exercise, apathy, older adults, non-pharmacological intervention, systematic review, meta-analysis

## Abstract

**Objectives:**

The objectives of this systematic review and meta-analysis were to evaluate the overall efficacy of physical exercise on apathy in older adults and to provide evidence for alleviating and improving apathy.

**Methods:**

This study was conducted following the PRISMA guidelines and the Cochrane Handbook for the Evaluation of Systems of Intervention. A comprehensive search was performed across databases, including Cochrane, EMBASE, PubMed, and Web of Science, with a cutoff date of January 2025. Data extraction, organization, and quality assessment were performed using appropriate software. Stata was used to analyze and process the data and test for publication bias.

**Results:**

The analysis included 9 RCTs involving a total of 356 participants. The meta-analysis revealed a significant improvement in apathy (SMD = −0.32; 95% CI −0.53 to −0.11; *p* < 0.01) after the physical exercise intervention.

**Conclusion:**

Physical exercise effectively alleviates and improves apathy in older adults. Physical exercise is characterized by low risk and high therapeutic benefits and can be used as an alternative or adjunct to medications for the treatment of apathy. Given its favorable safety and efficacy profile, physical exercise should be an important intervention in the treatment of apathy in older adults, while control of the intensity of exercise and supervision of the safety of the exercise process must be considered.

**Systematic Review Registration:**

https://www.crd.york.ac.uk/PROSPERO/view/CRD420251123484, CRD420251123484.

## 1 Introduction

Apathy is a behavioral condition marked by sustained reductions in goal-directed behavior, affect, and cognitive motivation; it is associated with abnormalities in the functioning of frontal–striatal neural circuits and is commonly observed in neurodegenerative diseases (e.g., dementia, Parkinson’s disease) and psychiatric disorders (1–4). Worldwide, apathy is prevalent in neurodegenerative disorders and is characterized by a high prevalence and persistence, with apathy occurring in approximately 50–70% of Alzheimer’s disease (AD) patients (5) and apathy occurring in approximately 40% of Parkinson’s disease (PD) patients (6, 7). Furthermore, patients with mild cognitive impairment who exhibit symptoms of apathy often lead to deteriorate into dementia (8). Key features of apathy are reduced goal-directed behavior (9), delayed emotional responses (2), decision-making difficulties, and impaired planning abilities (10). Indeed, older adults face many dilemmas caused by aging, including abnormal neurologic function (11), neurotransmitter imbalances (12), vascular and metabolic dysfunction (13), frustration triggered by the loss of function (2), social isolation (14), and other depressive comorbidities (15). The cumulative impact of these unfavorable factors may increase the risk of apathy in older adults. There is substantial evidence for the widespread occurrence of apathy symptoms within the aging population (2, 12, 14, 16, 17), and apathy is connected with a range of deficits and harms, including impaired of functioning in daily activities (3), accelerated deterioration of cognitive function (18), further increases in the burden of care (19), and increased patient mortality (20). These findings show that apathy not only seriously affects the physical and psychological health of the older adults and poses a serious challenge to their well-being in old age, but also imposes significant pressure on both society and families.

Several interventions have been introduced to assist older adults in successfully cope with stress and reducing apathy, including carboplatin (21), diethylpropion (22), methylphenidate (23), memantine, and several cholinesterase inhibitors (e.g., donepezil and rifampicin) (24), which are effective in treating apathy. However, long-term use of such medications can lead to a variety of side effects, such as gastrointestinal discomfort, cardiovascular risk, risk of care dependency, drowsiness, and even increased risk of pneumonia and stroke (24–26). In this context, improving apathy through physical exercise may help address these limitations. As a self-directed health behavior, exercise (hereafter referred to as “physical exercise”; as defined by the WHO, it is planned, structured, and repetitive physical activity undertaken to improve health, (27) encompassing modalities such as aerobic, resistance, balance training, as well as Tai Chi, dance, etc.) offers high participant engagement and low cost, advantages that are difficult to replicate with pharmacotherapy (28–31).

Although previous evidence suggests that physical exercise is effective in reducing the progression of apathy in older age groups, the conflicting results suggest that ambiguity remains regarding the outcome of physical exercise that improves apathy (12, 32–34). Several studies have demonstrated a lack of strong evidence for a direct link between physical exercise interventions and the alleviation of apathy in older adults (35, 36). As a result, the issue is still marked by differing expert perspectives as to whether the progression of apathy symptoms in the older population can be effectively mitigated and ameliorated. Notably, major recent trials have been conducted in this area. For example, Lautenschlager et al. (37) in 2023 reported a large RCT on physical activity in older adults, and the findings further support the benefits of exercise on cognitive health and related neuropsychiatric symptoms. This reflects the latest developments in interventions for late-life apathy and provides a cutting-edge context for our review. However, in published randomized controlled trials (RCTs) of physical exercise interventions for apathy, the scales for assessing apathy symptoms varied, resulting in different effect sizes (29–31, 38). For older adults facing multiple risk factors, managing apathy through physical exercise in place of pharmacological approaches may better support both psychological and physical health, along with overall life satisfaction (39, 40). Furthermore, recent systematic reviews and meta-analyses have documented positive effects of exercise on older adults’ mental health. For example, exercise interventions have been shown to significantly reduce depressive symptoms and improve cognitive function in older adults populations (41, 42). Therefore, given the potential of exercise to enhance overall psychological well-being in seniors, it is important to specifically investigate its impact on apathy, a distinct neuropsychiatric symptom.” This systematic review and meta-analysis aimed to determine the effectiveness of physical exercise on apathy in an older population and to provide evidence for alleviating and improving apathy.

## 2 Materials and methods

### 2.1 Search strategy

The study design and reporting were informed by the Cochrane Handbook for Systematic Reviews of Interventions and the PRISMA guidelines. The literature search was conducted across the following four databases: PubMed, Embase, Cochrane, and Web of Science, using both medical subject terms and free-text terms. The timeframe of the search was from the beginning of each database to January 2025. The search was conducted in four databases: PubMed, Embase, Cochrane, and Web of Science. Additionally, we searched grey literature databases (e.g., ProQuest Dissertations) and non-English databases such as the CNKI and J-STAGE to avoid missing key evidence. However, no additional randomized controlled trials meeting the inclusion criteria were identified. The search strategy for the PubMed database is shown in Table 1.

**Table 1:** Search strategy on PubMed.

Query number	Search terms
#1	Exercise [MeSH Terms]
#2	Exercise [Title/Abstract] OR Acute Exercise [Title/Abstract] OR Aerobic Exercise [Title/Abstract] OR Exercise Training [Title/Abstract] OR Exercise, Aerobic [Title/Abstract] OR Exercise, Isometric [Title/Abstract] OR Exercise, Physical [Title/Abstract] OR Isometric Exercise [Title/Abstract] OR Physical Activity [Title/Abstract] OR Resistance Training [Title/Abstract] OR Strength Training [Title/Abstract] OR Weight-Bearing Exercise Program [Title/Abstract] OR Weight-Bearing Strengthening Program [Title/Abstract] OR Weight-Lifting Exercise Program [Title/Abstract] OR Weight-Lifting Strengthening Program [Title/Abstract]
#3	#1 OR #2
#4	Randomized Controlled Trial [Publication Type]
#5	Randomized controlled trial [Title/Abstract] OR random [Title/Abstract] OR random* [Title/Abstract] OR placebo [Title/Abstract]
#6	#4 OR #5
#7	Apathy [Mesh]
#8	Apathy [Title/Abstract]
#9	#7 OR #8
#10	#3 AND #6 AND #9

The asterisk (*) was used as a wildcard to represent one or more characters, allowing for the retrieval of all variations of a root word.

### 2.2 Inclusion and exclusion criteria

The inclusion and exclusion criteria were established according to the PICOS framework. Eligible studies for this systematic review and meta-analysis met the following criteria: (P) Population: older adults; (I) Intervention: physical exercise interventions (e.g., aerobic training, resistance exercise, balance exercises, multimodal programs, yoga, Tai Chi, dance-based activities); (C) Control group: control conditions limited to routine care or standardized rehabilitation protocols; (O) Outcome: assessments of apathy symptoms and associated outcome measures; (S) Study design: randomized controlled trial.

Studies were excluded from this systematic review and meta-analysis based on the following PICOS criteria: (P) Population: patients under 65 years old population; (I) Intervention: non-exercise-based protocols (e.g., pharmacological treatments, psychotherapy-only approaches); (C) Control: inappropriate control conditions; (O) Outcome: studies lacking validated apathy metrics or related symptom assessments; (S) Study design: non-randomized controlled trials, e.g., quasi-experimental trials, study protocols, review articles, conference abstracts, editorial commentaries, etc.

### 2.3 Study selection and quality assessment

Dual independent reviewers (HJ and ZM) conducted literature screening using EndNote 20.6 reference management software, adhering to predefined eligibility criteria. Following the initial automated duplicate removal by the software, residual redundant entries were eliminated through manual verification. Subsequent title/abstract screening and full-text assessment were independently performed by both researchers. Discrepancies in study selection were resolved through iterative consensus discussions, with arbitration by a third investigator (JH) when required.

The risk of bias (ROB) was conducted utilizing Cochrane Review Manager 5.4, applying seven core criteria: (1) random sequence generation, (2) allocation concealment, (3) participant/personnel blinding, (4) outcome assessor blinding, (5) data completeness, (6) selective reporting, and (7) other biases. Each criterion received tripartite classification: Low, High, or Unclear risk. Studies received tiered risk stratification according to cumulative high-risk domains: High (≥5 criteria), Moderate (3–4 criteria), and Low (≤2 criteria).

### 2.4 Data extraction

Two independent investigators systematically extracted the following variables from each eligible study utilizing a standardized data collection template encompassing pre-specified domains: (1) basic information comprising investigator names, publication chronology, and geographic origin of studies; (2) subject characteristics, including the mean age and presence of other diseases; (3) experimental setup, including sample size, type of exercise, period, frequency, and duration; and (4) primary endpoint measurements and measurement tools.

### 2.5 Statistical analysis

Both the systematic review and meta-analysis focused exclusively on apathy as the outcome. Effect sizes were combined via standardized mean differences (SMDs) and 95% confidence intervals (CIs) due to differences in the measurement tools used in the different RCTs (43). For all meta-analyses, heterogeneity between studies was assessed via chi-square tests based on the *Q*-test and the *I*^2^ statistic, with a *p*-value <0.05 indicating statistical significance (44). According to the recommendations of the Cochrane Manual, significant heterogeneity existed when the *p*-value was <0.10 or *I*^2^ >50%, and the results were combined via a random effects model. Otherwise, when there was no significant heterogeneity (*p*-value >0.10 or *I*^2^ <50%), the results were combined using a fixed-effects model (43). To explore potential sources of heterogeneity, we conducted subgroup analyses and meta-regression. Statistical analyses were conducted using Stata 18.0.

## 3 Results

### 3.1 Literature selection

**Figure 1:**
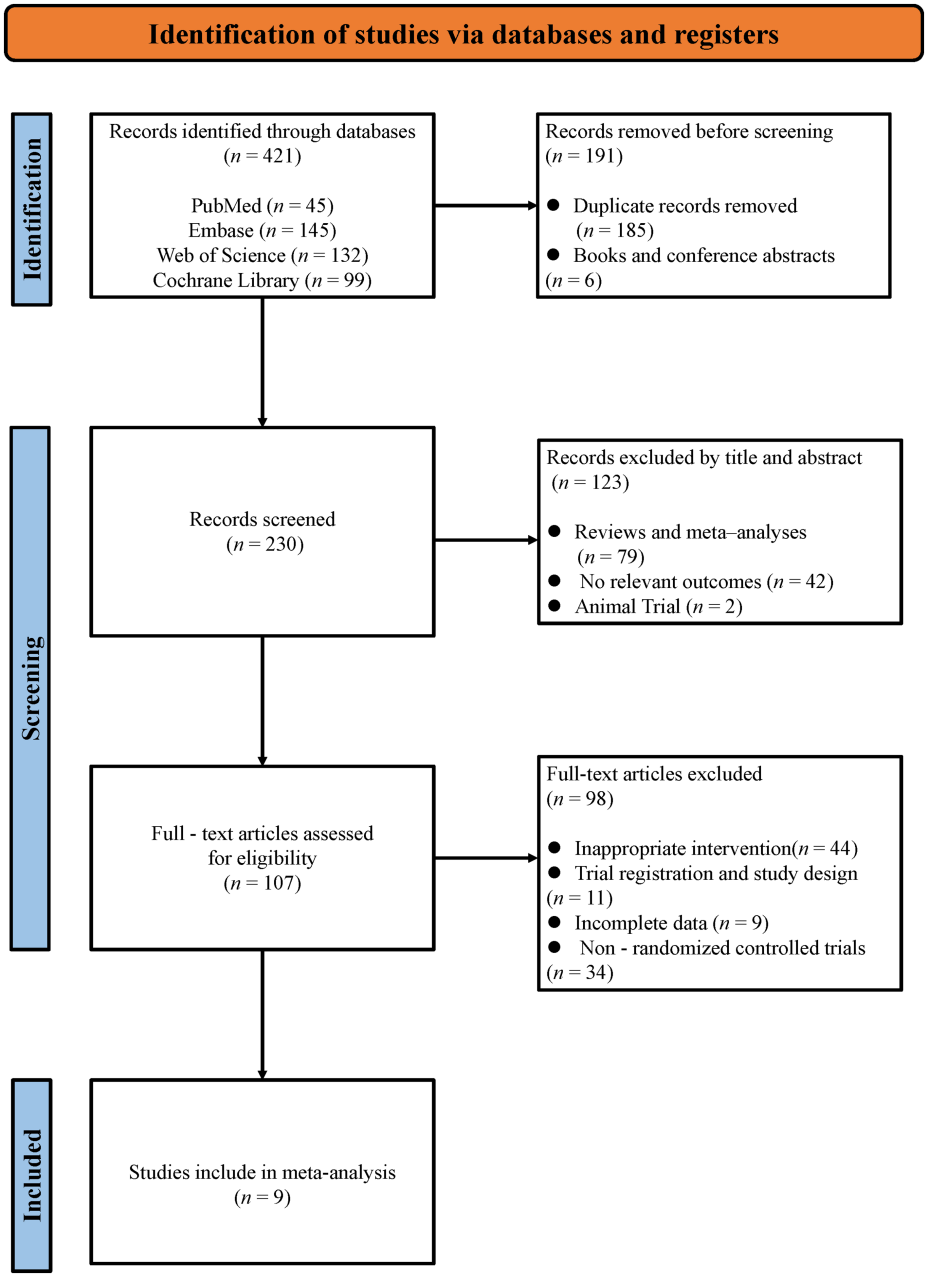
Flowchart of study selection.

The systematic search across four databases yielded 421 initial records. Following the removal of 185 duplicates and 6 non-journal materials (books/conference abstracts), 230 publications underwent title/abstract screening, which excluded 42 irrelevant studies, 79 secondary analyses (reviews/meta-analyses), and 2 non-human trials. The remaining 107 articles underwent full-text assessment.” After a careful and thorough assessment, 98 articles were excluded because of noncompliance with outcome metrics, non-randomized controlled trials, incomplete data, inappropriate article types (e.g., conference abstracts), or inappropriate intervention types. Finally, we included nine published randomized controlled trials in this systematic review and meta-analysis (28–31, 38, 45–48). The entire selection process is described in Figure 1.

### 3.2 Characterization of the study

Nine full-text RCTs met the inclusion criteria and were conducted within multiple countries. The study populations included older adults in Italy (3 studies), Japan (2 studies), Canada (1 study), Germany (1 study), Norway (1 study), and Australia (1 study). A total of 185 subjects were assigned to the intervention group, with mean ages ranging from 63.3 to 88.1 years, whereas 171 subjects were assigned to the control group, with mean ages ranging from 66.6 to 86.4 years. The duration of the intervention ranged from 4 weeks to 12 weeks, the frequency ranged from 1 to 4 times per week, and the duration ranged from 25 min to 120 min. The main characteristics of the nine randomized controlled trials are shown in Table 2.

**Table 2:** The main characteristics of included randomized controlled trials.

Included studies	Population	Age [Mean(SD)]	Total/M%	Intervention	Control
Cugusi et al. (38) (Italy)	Sardinian outpatients with idiopathic Parkinson’s disease	T: 68.10 ± 8.70 C: 66.60 ± 7.30	T: 10/80% C: 10/80%	Nordic Walking program: Length: 12 weeks Freq: 2 times a week Duration: 60 min	Routine care
D’Cunha et al. (45) (Australia)	Residents with cognitive impairment from a 72—bed high—care residential aged—care facility	T: NR C: NR	T: 5/NR C: 5/NR	Virtual cycling experience (VCE): Length: 7 weeks Freq: 1 time a week Duration: 25 min	Routine sitting and lying activities
Hashimoto et al. (46) (Japan)	Mild—moderate Parkinson’s disease patients from local PD patient associations	T: 67.90 ± 7.00 C: 69.70 ± 4.00	T: 15/25% C: 14/50%	Dance intervention: Length: 12 weeks Freq: 1 time a week Duration: 60 min	Non-intervention
Sacheli et al. (28) (Canada)	Participants with mild to moderate (Hoehn & Yahr stages I-III) idiopathic Parkinson’s disease	T: 66.76 ± 5.98 C: 67.85 ± 8.50	T: 20/65% C: 15/60%	Aerobic exercise intervention: Length: 3 months Freq: 3 times per week Duration: 40–60 min	Stretching
Schaible et al. (47) (Germany)	Non-demented Parkinson’s disease patients (mild to moderate)	T: 63.29 ± 8.48 C: 65.50 ± 8.21	T: 14/50% C: 12/83%	Amplitude-specific movements Length: 4 weeks Freq: 4 times a week Duration: 60 min	Routine care
Solla et al. (48) (Italy)	Parkinson’s disease patients from the outpatient Movement Disorders Clinic of the University of Cagliari	T: 67.40 ± 6.10 C: 67.40 ± 6.10	T: 10/60% C: 10/70%	Sardinian folk dance (Ballu Sardu, BS) program: Length: 12 weeks Freq: 2 times a week Duration: 90 min	Routine care
Tanaka et al. (29) (Japan)	Older adult patients with dementia in a geriatric health service facility	T: 88.10 ± 8.10 C: 84.20 ± 7.40	T: 16/37.5% C: 15/46.7%	Group-based intervention: Length: 8 weeks Freq: 2 times a week Duration: 45 min	Routine care
Telenius et al. (30) (Norway)	Nursing home residents with dementia from 18 nursing homes	T: 86.9 ± 7.00 C: 86.4 ± 7.80	T:82/28% C:81/27%	High-Intensity Functional Exercises (HIFE) program: Length: 12 weeks Freq: 2 times a week Duration: 50–60 min	Leisure activities
Vitale et al. (31) (Italy)	Non-demented Parkinson’s disease patients	T: NR C: NR	T: 14/NR C: 14/NR	Biodanza SRT program, combining movement, music, and group interaction Length: 12 weeks Freq: 1 time a week Duration: 120 min	No-intervention

NR, not reported in the original study.

### 3.3 Quality assessment

**Figure 2:**
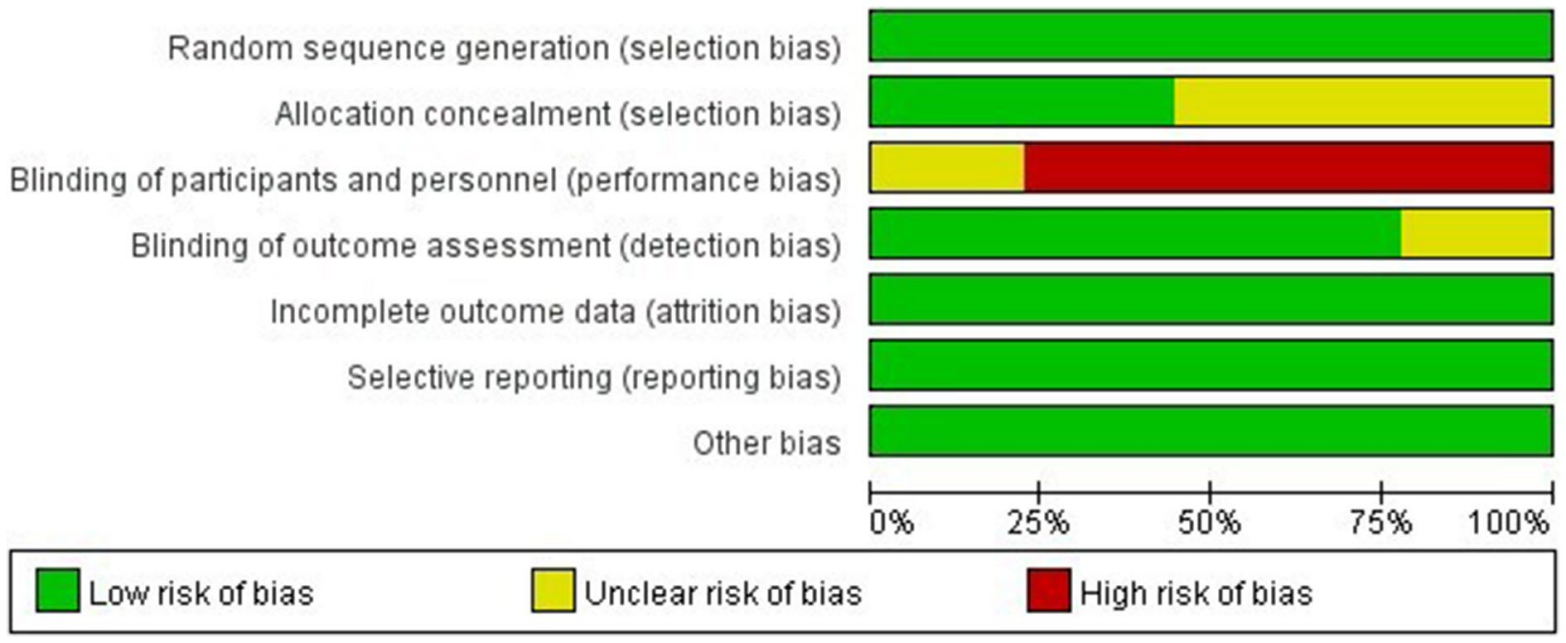
Risk of bias graph.

**Figure 3:**
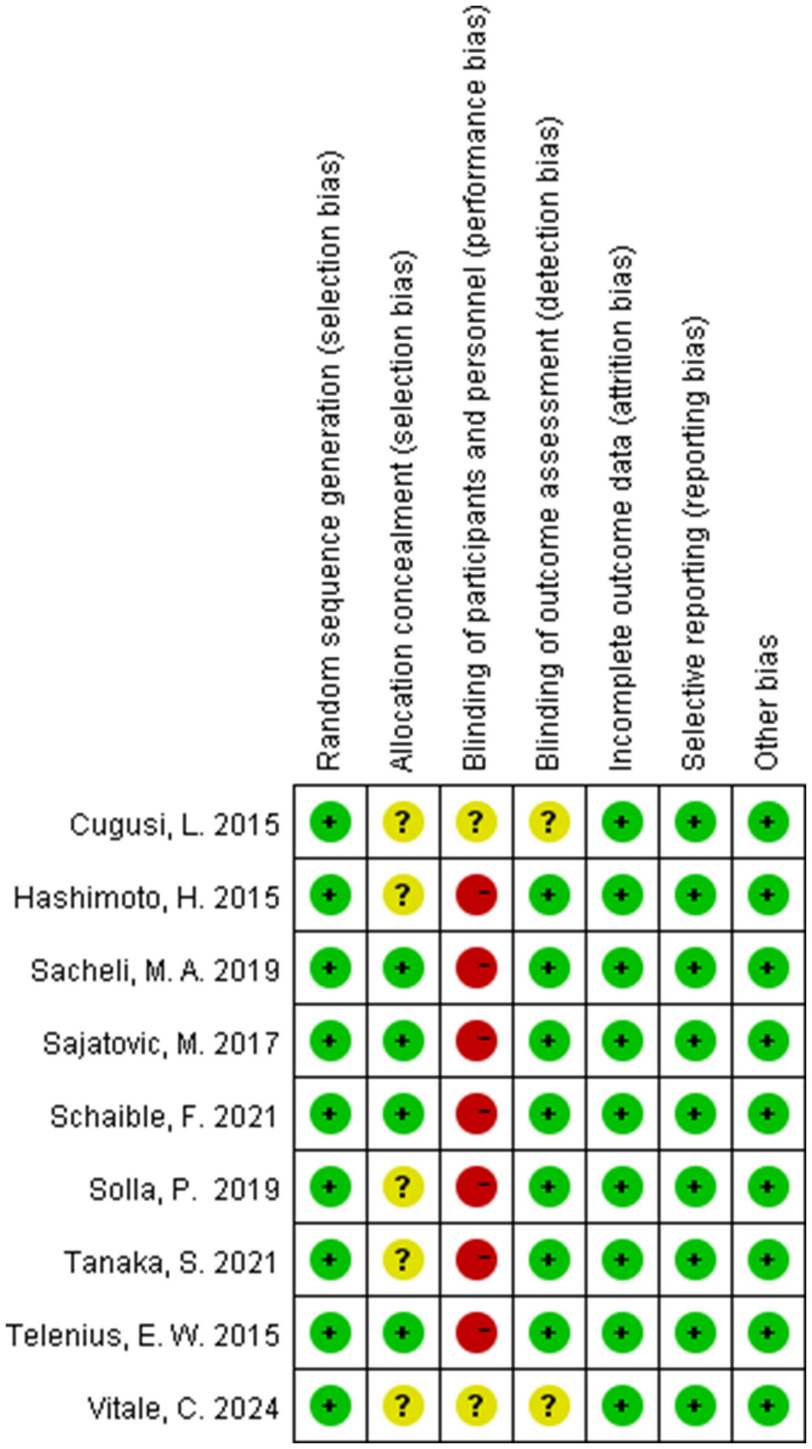
Risk of bias summary.

All nine RCTs explicitly documented randomization procedures using computerized systems or random number tables, with four trials employing sealed opaque envelopes for allocation concealment. Seven studies blinded the assessors of the study outcomes. Data completeness was generally good for all nine studies, with no evidence of selective reporting observed, and none were otherwise biased. The detailed quality assessment results are shown in Figures 2, 3.

### 3.4 Meta-analysis

**Figure 4:**
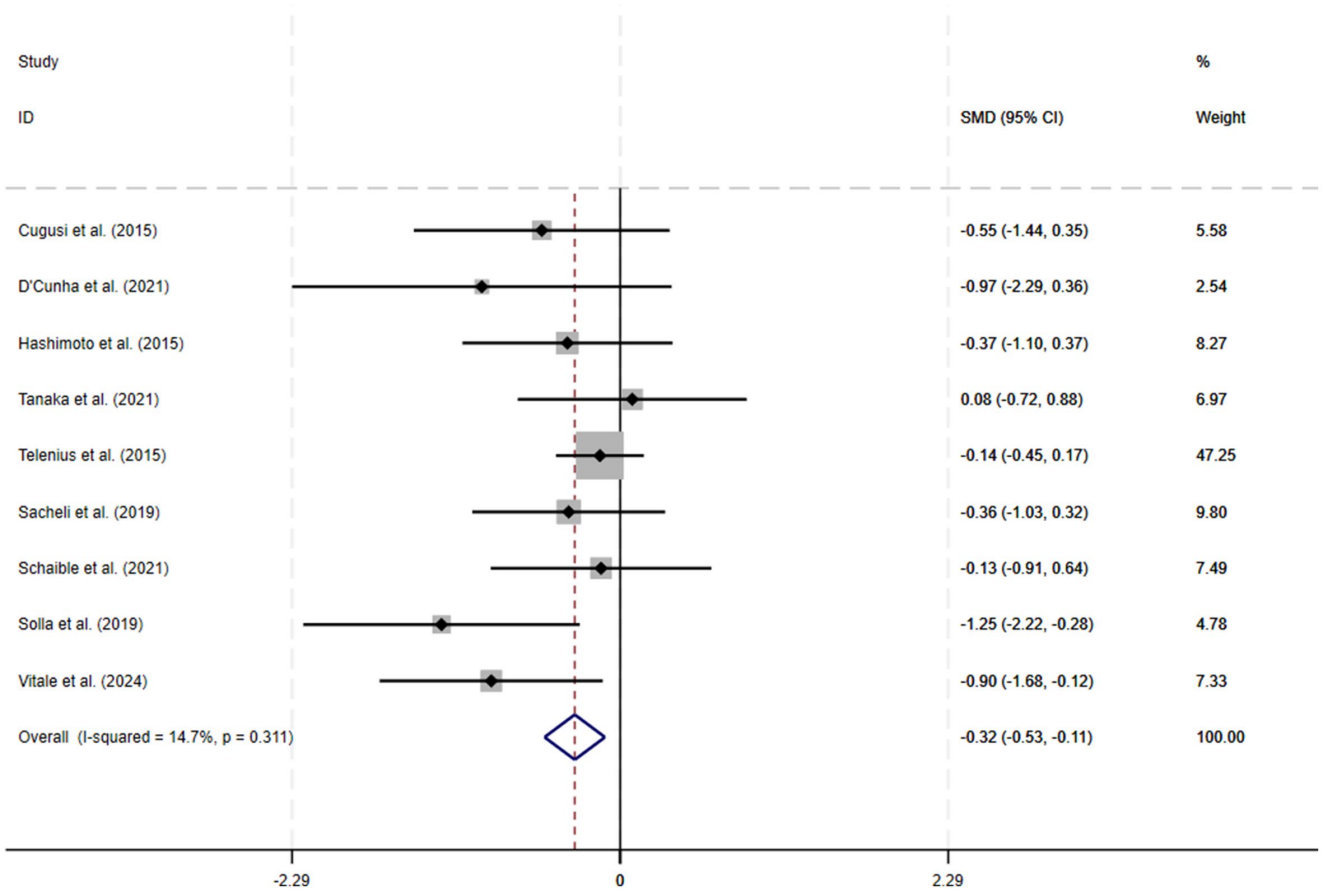
Forest plot of the effects of physical exercise on apathy.

A total of 9 RCTs were included in the meta-analysis, and heterogeneity was examined via a chi-square test based on the *I*^2^ statistic, which indicated that apathy (*I*^2^ = 14.7%; *p* = 0.31) showed no significant heterogeneity; therefore, a fixed-effects model was chosen for the meta-analysis. The results of the meta-analysis revealed a significant improvement in apathy (SMD = −0.32; 95% CI −0.53 to −0.11; *p* < 0.01). A forest plot of the meta-analysis results for the outcome variables is shown in Figure 4.

### 3.5 Sensitivity analysis

Sensitivity analyses assessed the effect of each study on the combined effect size by excluding it one by one. The results showed that after excluding any single study, the effect size estimates ranged from −0.27 to −0.48, and all 95% confidence intervals did not contain zero values (−0.052 to −0.767), suggesting that the statistical significance of the combined results was not significantly affected and that the overall results were robust. In particular, after excluding the study performed by Telenius et al. (30), the effect size estimate (−0.476) was more negative than the combined results (−0.318), but its confidence intervals (−0.767 to −0.185) still partially overlapped with the combined intervals (−0.529 to −0.106), suggesting that the study had an impact on the strength of the effect size, but did not change the effect’s direction or significance level. The remaining studies excluded estimates that fluctuated less (−0.27 to −0.35), further supporting the stability of the results. In summary, the sensitivity analysis confirmed the reliability of the findings of this study. The outcome-specific results of the sensitivity analyses are shown in Table 3.

**Table 3:** Sensitivity analysis for outcomes by omitting individual studies.

Study omitted	SMD	95% CI	
		Lower bound	Upper bound
Cugusi et al. (38)	−0.3	−0.52	−0.09
D’Cunha et al. (45)	−0.3	−0.52	−0.09
Hashimoto et al. (46)	−0.31	−0.53	−0.09
Sacheli et al. (28)	−0.31	−0.54	−0.09
Schaible et al. (47)	−0.33	−0.55	−0.11
Solla et al. (48)	−0.27	−0.49	−0.05
Tanaka et al. (29)	−0.35	−0.58	−0.13
Telenius et al. (30)	−0.48	−0.77	−0.19
Vitale et al. (31)	−0.27	−0.49	−0.05

SMD = Standard Mean Difference; CI = Confidence Interval.

### 3.6 Subgroup analyses and Meta-regression results

The meta-regression revealed that none of the examined variables—including exercise modality (single-mode vs. multi-modal), participant population (Parkinson’s disease vs. cognitive impairment), intervention duration (≥8 weeks vs. ≤12 weeks), or study region (Europe vs. other)—had a significant impact on the intervention effect (all *p* > 0.05). Consistently, the subgroup analysis showed that although there were some differences in effect sizes between categories (multi-modal exercise interventions produced a more pronounced improvement in apathy symptoms, SMD = −0.55, 95% CI −0.95 to −0.15, compared to single-mode exercise, SMD = −0.23, 95% CI −0.48 to 0.02; and interventions in Parkinson’s disease patients had a greater effect, SMD = −0.54, 95% CI −0.86 to −0.23, than those in cognitively impaired patients, SMD = −0.15, 95% CI −0.43 to 0.13), none of these differences reached statistical significance (between-group *p* = 0.19 and 0.08, respectively). Similarly, 12-week interventions (SMD = −0.35, 95% CI −0.58 to −0.12) did not show a significantly greater effect than 8-week interventions (SMD = −0.17, 95% CI −0.68 to 0.34; *p* = 0.53), nor was there a significant difference in effect between studies conducted in Europe versus other regions (*p* = 0.95). These findings suggest that the low heterogeneity observed in our analysis may be attributable to a relatively high homogeneity in participant characteristics and intervention protocols across the included studies, and no specific moderating factors were identified as significantly influencing the effect size. The detailed results are presented in Tables 4, 5.

**Table 4:** Meta-regression analyses of sources of heterogeneity.

Variables	*k*	*β*	SE	*t*	95% CI		*p*-value
					Lower bound	Upper bound	
Exercise type	9	−0.263	0.302	−0.87	−1.101	0.575	0.433
Diseases type	9	0.341	0.261	1.31	−0.384	1.067	1.310
Intervention duration	9	−0.164	0.328	−0.50	−1.075	0.748	0.644
continent	9	0.183	0.299	0.61	−0.648	1.014	0.574

*k* = Number of trials; *β* = Regression coefficient; SE = Standard error; CI = Confidence interval.

**Table 5:** Subgroup analyses of sources of heterogeneity.

Variables	Subgroup	SMD	95% CI		*p*-value (SMD)	Heterogeneity			Group differences (*p*-value)
			Lower bound	Upper bound		*I*^2^ (%)	*p*-value	*Z*	
Exercise type	Single-mode exercise	−0.23	−0.48	0.02	0.07	0.00	0.70	1.82	0.19
	Multimodal exercise	−0.55	−0.95	−0.15	<0.01	44.80	0.14	2.67	
Diseases type	Parkinson’s disease	−0.54	−0.86	−0.22	<0.01	0.00	0.49	3.27	0.08
	Cognitive impairment	−0.15	−0.43	0.13	0.29	0.00	0.41	1.05	
Intervention duration	12 weeks	−0.35	−0.58	−0.12	<0.01	30.60	0.21	2.94	0.53
	8 weeks	−0.17	−0.68	0.34	0.51	0.00	0.41	0.65	
continent	Europe	−0.32	−0.57	−0.07	0.01	46.40	0.11	2.54	0.95
	Other	−0.31	−0.53	−0.11	0.14	0.00	0.59	1.49	

SMD, Standardized mean difference; CI, Confidence interval; *I*^2^, Heterogeneity index in percentage (range: 0–100%).

### 3.7 Publication bias testing

**Figure 5:**
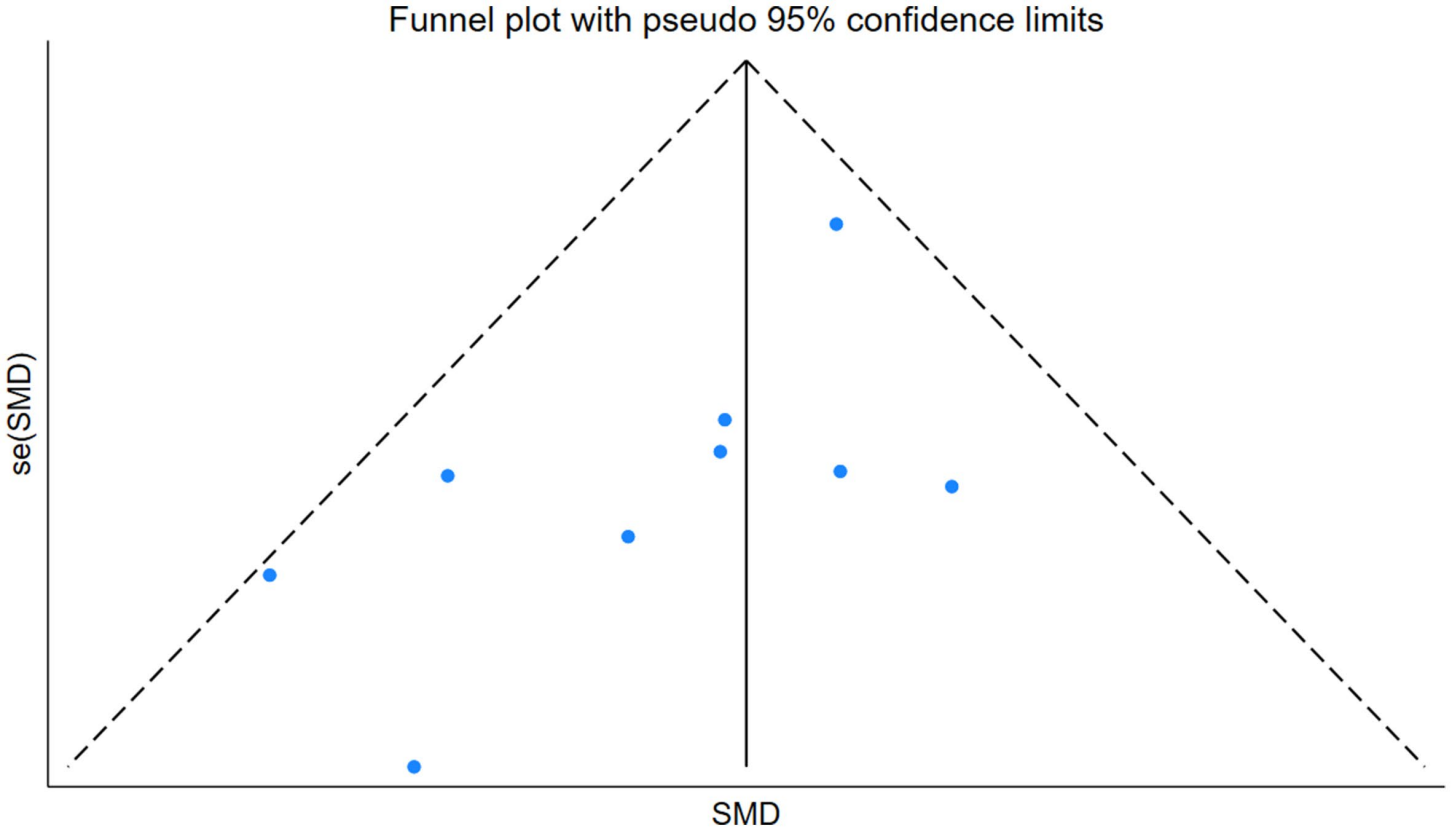
Funnel plot for meta-analysis.

We used Stata software version 18 to generate outcome funnel plots to assess potential publication bias. The results revealed that the symmetrical distribution of all studies in the funnel plot could not be well observed, so we further performed Egger’s test to quantitatively analyze the publication bias. The Egger’s test yielded a *p*-value of 0.066, and Begg’s test yielded 0.076. Although neither reached statistical significance by conventional standards, the Egger’s *p*-value is very close to the 0.05 threshold, indicating a borderline risk of publication bias; therefore, the results should be interpreted with caution. The funnel plot is shown in Figure 5.

## 4 Discussion

Alleviating apathy symptoms in middle-aged and older adult patients through targeted physical exercise interventions is critical for slowing down the disease process and improving quality of life, as apathy is not only one of the core manifestations of neuropsychiatric symptoms (49) but also associated with a variety of adverse outcomes, notably accelerated cognitive decline (50), a diminished capacity for daily functioning, leading to a lower quality of life, a greater risk of developing dementia (51) and an increased cost and burden of care (52). This systematic review and meta-analysis found that physical exercise significantly improves apathy symptoms in older adults. Given the low risk and high efficacy of exercise interventions, our findings further support the integration of multi-modal exercise programs as a non-pharmacological treatment for geriatric apathy in clinical practice (41). Future research should focus on identifying the optimal exercise dose and modalities, and on examining the effects in different subpopulations (e.g., those with cognitive impairment or comorbid conditions), to enhance the specificity and effectiveness of such interventions.” The results revealed that patients in the exercise intervention group experienced a significant reduction in apathy symptoms compared with those in the control group, with a combined effect size of standardized mean difference (SMD = −0.32, 95% CI −0.53 to −0.11, *p* < 0.01). Although the pooled effect size corresponds to only a low-to-moderate magnitude of improvement (SMD ≈ 0.32, a small effect by Cohen’s criteria), this improvement still holds significance in clinical terms. For example, on the Apathy Evaluation Scale (AES), a reduction of about 3–4 points is considered the threshold for a clinically important improvement (53). By that measure, the SMD = −0.32 observed in our study is roughly equivalent to this threshold, suggesting that the improvement in apathy symptoms due to exercise may be tangible to patients. It is noteworthy that apathy is often difficult to treat – even a modest improvement can appreciably enhance a patient’s engagement in daily activities and emotional drive. Furthermore, given that exercise interventions are very safe with minimal side effects, any degree of clinical benefit they confer should be valued. This finding further supports the feasibility of non-pharmacologic interventions in geriatric mental health management, especially for patients with symptoms of apathy that are difficult to ameliorate directly with medication.

Among the nine included RCTs, methodological limitations were present: only four reported allocation concealment, and two lacked blinding of outcome assessors. This absence of blinding could bias results, particularly for subjective outcomes such as apathy. However, sensitivity analyses excluding unblinded studies showed that the effect of exercise on apathy remained significant and consistent, indicating that our main findings are robust despite some risk of bias (54). Nonetheless, future trials should adopt rigorous randomization and blinding procedures to strengthen the evidence and minimize potential bias. In addition, the overall heterogeneity in our meta-analysis was low (I^2^ = 14.7%), and we believe this is largely attributable to the high homogeneity of the included studies. Most of the studies were conducted in similar settings (6 of the 9 studies were based in Europe), and the participant populations and intervention protocols were also comparable across studies (for example, several studies focused on patients with Parkinson’s disease and employed exercise programs of similar types and intensities). This high degree of similarity likely led to consistent effect directions and magnitudes, thereby reducing between-study heterogeneity.

Indeed, the mechanism of action of physical exercise interventions to improve apathy symptoms in middle-aged and older adults can be analyzed from multiple dimensions. At the physiological level, physical exercise may optimize the neural basis of motivational decision-making by modulating the dopaminergic system. Midbrain limbic dopamine circuits (e.g., the nucleus ambiguous-prefrontal pathway) play a central role in the “reward-effort trade-off,” and dopamine depletion in the nucleus ambiguous leads to a low-effort selection bias, whereas regular exercise enhances behavioral engagement in high-effort goals by promoting striatal dopamine release of behavioral engagement (1, 55). Furthermore, exercise promotes neuroplasticity and synaptic function in the hippocampus and prefrontal cortex by increasing the levels of brain-derived neurotrophic factor (BDNF) and insulin-like growth factor (IGF-1) (56, 57), which are correlated with increased hippocampal volume and increased BDNF and BDNF release in Alzheimer’s disease patients. This finding is consistent with research evidence that increased hippocampal volume and BDNF levels are negatively associated with apathy symptoms in Alzheimer’s disease patients (58, 59). Moreover, by improving brain endothelial function and oxidative stress regulation, exercise can increase the oxygen supply to motivation-related brain regions (e.g., the anterior cingulate gyrus and ventral striatal body) and alleviate neurological impairments caused by chronic hypoxia (56, 60). At the level of psychosocial mechanisms, individualized versus structured exercise regimens work through dual pathways—individualized exercise choices (e.g., interest-oriented types of exercise)to enhance behavioral engagement (3), whereas group exercise (e.g., Tai Chi/dance) activates endogenous engagement through social feedback (e.g., peer motivation or group exercise) activates the endogenous reward system (61, 62). In contrast, cognitive stimulation combined with exercise training, for example, rebuilds behavioral motivation through the goal-attainment-positive-feedback cycle and reduces behavioral avoidance triggered by a low reward threshold (3, 63). Functional imaging evidence further reveals that exercise enhances functional integration of the posterior cingulate gyrus with the supplementary motor region, facilitating the behavioral transition from “intention assessment” to “action execution” (60), and that this enhanced neural efficiency of the prefrontal–basal ganglia circuitry provides a cross-mechanically integrated explanatory framework for the improvement of apathy by exercise.

Building on these findings, we further elucidated the underlying mechanisms by conducting a population-based analysis. In neurodegenerative diseases such as Parkinson’s disease, apathy is mainly caused by neurobiological dysfunction, particularly deficits in dopaminergic pathways. Evidence shows that regular exercise can enhance striatal dopamine release and reward-related brain activity, partially compensating for motivational deficits (28, 64). Neuroimaging studies confirm that exercise increases dopamine transporter availability and striatal activation in these patients. In contrast, apathy among healthy older adults is more often related to mild age-related brain changes and psychosocial factors, such as decreased social interaction after retirement. In this population, exercise relieves apathy mainly by promoting brain health, upregulating neurotrophic factors like BDNF and IGF-1, and enhancing synaptic plasticity in the hippocampus and prefrontal cortex (65, 66). These observations illustrate that the mechanisms of exercise’s anti-apathy effects may differ between populations: in Parkinson’s patients, the key is restoring dopaminergic function, whereas in other older adults, the benefits derive more from neurotrophic support and psychosocial stimulation. These distinctions highlight the importance of tailored exercise interventions according to the specific etiology of apathy.

This meta-analysis demonstrates that physical exercise has a positive impact on symptom improvement in older adults apathetic patients after the intervention, compared with controls, and that the results vary across different types of physical exercise. Based on our findings, we suggest that clinicians may consider multi-modal exercise programs as one strategy to manage apathy. For instance, combining aerobic, resistance, and balance training or incorporating elements like Tai Chi and dance into exercise, along with some cognitive stimulation, could potentially improve older adults’ motivation and emotional engagement through multiple pathways. However, since current evidence directly comparing multi-modal versus single-modal exercise is limited (our subgroup analysis did not observe a significant difference), we cannot yet conclude that multi-modal exercise is definitively superior to single-mode exercise. In particular, only one study in our meta-analysis employed a combined physical and cognitive intervention; therefore, any assertion of a relative advantage of multimodal approaches should be viewed with caution. We recommend that future RCTs directly compare different exercise modalities in their effect on apathy to inform optimal intervention strategies. Until more evidence is available, multi-modal exercise can be considered a promising approach to explore, but it is not the only option. The outcomes of this research support the use of physical exercise as a non-pharmacological intervention for older adult patients with apathy, providing a preliminary evidence-based basis for the development of individualized exercise programs in clinical practice and highlighting the need to further optimize the dosages and types and type of interventions in future studies.

It should be noted that some individual studies did not find a significant benefit of exercise interventions. For instance, Hashimoto et al. (46) reported that dance intervention in older patients with Parkinson’s disease did not yield a significant improvement in apathy, as the 95% confidence interval included the line of no effect. This highlights that factors such as cultural context and intervention modality may influence outcomes; for example, dance may be less accepted or engaging in the studied Japanese population. Variability in participants’ baseline characteristics and intervention adherence may also contribute to inconsistent results. Thus, while our overall findings support the efficacy of exercise interventions, we remain cautious regarding these potential sources of heterogeneity and recommend that future studies further investigate the roles of cultural factors and intervention types in mediating exercise effects on apathy. These observations highlight the imperative for methodological enhancements, including expanded participant cohorts and refined study designs, to improve the precision of evaluating sustained physical exercise effects on target outcomes. In addition, the optimal duration of physical exercise interventions remains unclear, despite some RCTs limiting the intervention duration to 40–60 min. This ambiguity means that the ‘dose–response’ relationship between physical exercise interventions and outcomes related to apathy relief is unclear. Similarly, the timing of physical exercise interventions remains unclear [cortisol levels peak in the morning (67), whereas evening exercise may indirectly reduce apathy by improving sleep quality]. Do apathy symptoms rebound after cessation of a physical exercise intervention? These questions need to be further investigated in future studies to evaluate the relative effectiveness of various intervention modalities in alleviating apathy symptoms to optimize specific implementation strategies for physical exercise interventions. Finally, considering that most patients with apathy symptoms also suffer from other diseases, such as Alzheimer’s disease, Parkinson’s disease, and Huntington’s disease (68–70), and that they are older, resulting in varying degrees of physical functioning, it is important to consider that the implementation of the intervention may be more effective in alleviating apathy symptoms. Therefore, safety considerations must be taken into account when interventions are implemented. Appropriate protective measures should be taken to ensure the safety of interventions to minimize potential health risks.

In conclusion, this study indicates that physical exercise exerts a notably beneficial impact on apathy symptoms in older adults. The physical functions and social participation of the older adults may decline over the years, and during this period, the older adults may suffer from various illnesses and face difficulties such as a lack of social participation, the cumulative effect of which may lead to the emergence or worsening of symptoms of indifference. The cumulative effect of this distress and hardship may lead to the development or worsening of symptoms of apathy, which may, in turn, lead to the development of other psychological disorders. Body movement interventions, which are characterized by low risk and high therapeutic benefit, can be used as alternative or adjunctive approaches to treating apathy symptoms. Therefore, considering the advantages of physical exercise in terms of safety and efficacy, physical exercise interventions can be considered important interventions for the treatment of apathy symptoms in the geriatric population. Appropriate medication may be used on a case-by-case basis to further increase the therapeutic effect, and apathy symptoms can be significantly improved with this comprehensive therapeutic strategy of older adults.

## 5 Limitation

The current meta-analysis has several limitations. First, only 9 RCTs (comprising 356 total participants) were included in our analysis, and this relatively small sample size may have limited the statistical power to detect small effects. As a result, even though the sensitivity analysis showed that the pooled effect remained statistically significant and robust after excluding any single study (with estimated SMDs ranging from −0.27 to −0.48). This finding underscores the scarcity of research on exercise interventions for apathy in the older adults, highlighting the need for more high-quality, large-sample RCTs in the future to verify the conclusions of our study. In addition, none of the RCTs included in our review performed analyses adjusting for this comorbidity. This implies that the effect of exercise on apathy might be influenced by concurrent depressive symptoms. For instance, if participants were also depressed, an improvement in depression might partly account for the observed improvement in apathy. Due to limitations in the available data, we could not quantify the impact of depression as a confounder in our results. We therefore acknowledge the lack of control for co-morbid depression as a limitation of our study. Future research should assess and report participants’ depression levels and control for them in the analysis to clarify whether the apathy-reducing effect of exercise is independent of its effects on depression. Secondly, the exercise interventions in the included trials ranged from 4 to 12 weeks in duration; thus, no data are available on longer interventions. This limitation means we cannot determine whether the benefits of exercise are sustained after the intervention period—apathy symptoms might worsen again once exercise ceases. Finally, the trials employed different apathy assessment scales (e.g., the Starkstein Apathy Scale, Apathy Evaluation Scale, etc.), which may have different scoring criteria and sensitivity; this could further contribute to heterogeneity and affect the pooled effect estimate, and thus should be considered when interpreting the results. Future studies should increase the intervention duration and extend the follow-up period to explore whether a longer intervention can yield more enduring improvements in apathy and whether symptoms might recur after stopping the exercise. Prolonged observation will help evaluate the role of exercise interventions in the long-term management of apathy symptoms.

## 6 Conclusion

This systematic review and meta-analysis demonstrated that physical exercise interventions effectively improve apathy symptoms in older adults. Given its favorable safety profile and minimal side effects, physical exercise is recommended as an important treatment approach for geriatric apathy, provided that exercise intensity is appropriately managed and supervised.

## Data Availability

The original contributions presented in the study are included in the article/Supplementary material, further inquiries can be directed to the corresponding author.

## References

[1] Levy R Dubois B. Apathy and the functional anatomy of the prefrontal cortex-basal ganglia circuits. Cereb Cortex. (2006) 16:916–28. doi: 10.1093/cercor/bhj043, PMID: 16207933

[2] Marin RS. Apathy: a neuropsychiatric syndrome. J Neuropsychiatry Clin Neurosci. (1991) 3:243–54. doi: 10.1176/jnp.3.3.243, PMID: 1821241

[3] Massimo L Kales HC Kolanowski A. State of the science: apathy as a model for investigating behavioral and psychological symptoms in dementia. J Am Geriatr Soc. (2018) 66:S4–s12. doi: 10.1111/jgs.15343, PMID: 29659001 PMC5905718

[4] Starkstein SE Ingram L Garau ML Mizrahi R. On the overlap between apathy and depression in dementia. J Neurol Neurosurg Psychiatry. (2005) 76:1070–4. doi: 10.1136/jnnp.2004.052795, PMID: 16024880 PMC1739766

[5] Mulin E Leone E Dujardin K Delliaux M Leentjens A Nobili F . Diagnostic criteria for apathy in clinical practice. Int J Geriatr Psychiatry. (2011) 26:158–65. doi: 10.1002/gps.2508, PMID: 20690145

[6] Béreau M van Waes V Servant M Magnin E Tatu L Anheim M. Apathy in Parkinson’s disease: clinical patterns and neurobiological basis. Cells. (2023) 12:1599. doi: 10.3390/cells12121599, PMID: 37371068 PMC10297386

[7] D'Iorio A Maggi G Vitale C Trojano L Santangelo G. “Pure apathy” and cognitive dysfunctions in Parkinson’s disease: a meta-analytic study. Neurosci Biobehav Rev. (2018) 94:1–10. doi: 10.1016/j.neubiorev.2018.08.004, PMID: 30114389

[8] Sperling RA Aisen PS Beckett LA Bennett DA Craft S Fagan AM . Toward defining the preclinical stages of Alzheimer’s disease: recommendations from the National Institute on Aging-Alzheimer’s Association workgroups on diagnostic guidelines for Alzheimer’s disease. Alzheimers Dement. (2011) 7:280–92. doi: 10.1016/j.jalz.2011.03.003, PMID: 21514248 PMC3220946

[9] Glenn MB Burke DT O'Neil-Pirozzi T Goldstein R Jacob L Kettell J. Cutoff score on the apathy evaluation scale in subjects with traumatic brain injury. Brain Inj. (2002) 16:509–16. doi: 10.1080/02699050110119132, PMID: 12119086

[10] Radakovic R Abrahams S. Developing a new apathy measurement scale: dimensional apathy scale. Psychiatry Res. (2014) 219:658–63. doi: 10.1016/j.psychres.2014.06.010, PMID: 24972546

[11] Parrotta I Cacciatore S D’Andrea F D’Anna M Giancaterino G Lazzaro G . Prevalence, treatment, and neural correlates of apathy in different forms of dementia: a narrative review. Neurol Sci. (2024) 45:1343–76. doi: 10.1007/s10072-023-07197-7, PMID: 38015288 PMC10942903

[12] Mehak SF Shivakumar AB Saraf V Johansson M Gangadharan G. Apathy in Alzheimer’s disease: a neurocircuitry based perspective. Ageing Res Rev. (2023) 87:101891. doi: 10.1016/j.arr.2023.101891, PMID: 36871779

[13] Ligthart SA Richard E Fransen NL Eurelings LS Beem L Eikelenboom P . Association of vascular factors with apathy in community-dwelling elderly individuals. Arch Gen Psychiatry. (2012) 69:636–42. doi: 10.1001/archgenpsychiatry.2011.1858, PMID: 22664551

[14] Yang Q Wang Y Yang M Ge S Cheng S Wang C . Apathy co-occurs with subjective cognitive decline among community-dwelling older adults. Geriatr Nurs. (2022) 48:177–82. doi: 10.1016/j.gerinurse.2022.09.018, PMID: 36257223

[15] Geda YE Roberts RO Mielke MM Knopman DS Christianson TJH Pankratz VS . Baseline neuropsychiatric symptoms and the risk of incident mild cognitive impairment: a population-based study. Am J Psychiatry. (2014) 171:572–81. doi: 10.1176/appi.ajp.2014.13060821, PMID: 24700290 PMC4057095

[16] Gilmore-Bykovskyi A Block L Johnson R Goris ED. Symptoms of apathy and passivity in dementia: a simultaneous concept analysis. J Clin Nurs. (2019) 28:410–9. doi: 10.1111/jocn.14663, PMID: 30184283 PMC6326867

[17] Teixeira AL Gonzales MM de Souza LC Weisenbach SL. Revisiting apathy in Alzheimer’s disease: from conceptualization to therapeutic approaches. Behav Neurol. (2021) 2021:6319826. doi: 10.1155/2021/6319826, PMID: 34394772 PMC8356015

[18] Ruthirakuhan M Herrmann N Vieira D Gallagher D Lanctôt KL. The roles of apathy and depression in predicting Alzheimer disease: a longitudinal analysis in older adults with mild cognitive impairment. Am J Geriatr Psychiatry. (2019) 27:873–82. doi: 10.1016/j.jagp.2019.02.003, PMID: 30910421 PMC6646066

[19] Norton LE Malloy PF Salloway S. The impact of behavioral symptoms on activities of daily living in patients with dementia. Am J Geriatr Psychiatry. (2001) 9:41–8. doi: 10.1097/00019442-200102000-00007, PMID: 11156751

[20] Okura T Plassman BL Steffens DC Llewellyn DJ Potter GG Langa KM. Neuropsychiatric symptoms and the risk of institutionalization and death: the aging, demographics, and memory study. J Am Geriatr Soc. (2011) 59:473–81. doi: 10.1111/j.1532-5415.2011.03314.x, PMID: 21391937 PMC3088883

[21] Lazcano-Ocampo C Wan YM van Wamelen DJ Batzu L Boura I Titova N . Identifying and responding to fatigue and apathy in Parkinson’s disease: a review of current practice. Expert Rev Neurother. (2020) 20:477–95. doi: 10.1080/14737175.2020.1752669, PMID: 32290717

[22] Dolphin H Dyer AH McHale C O'Dowd S Kennelly SP. An update on apathy in Alzheimer’s disease. Geriatrics (Basel). (2023) 8:75. doi: 10.3390/geriatrics8040075, PMID: 37489323 PMC10366907

[23] Mintzer J Lanctôt KL Scherer RW Rosenberg PB Herrmann N van Dyck CH . Effect of methylphenidate on apathy in patients with Alzheimer disease: the ADMET 2 randomized clinical trial. JAMA Neurol. (2021) 78:1324–32. doi: 10.1001/jamaneurol.2021.3356, PMID: 34570180 PMC8477302

[24] Azhar L Kusumo RW Marotta G Lanctôt KL Herrmann N. Pharmacological management of apathy in dementia. CNS Drugs. (2022) 36:143–65. doi: 10.1007/s40263-021-00883-0, PMID: 35006557

[25] Masdrakis VG Markianos M Baldwin DS. Apathy associated with antidepressant drugs: a systematic review. Acta Neuropsychiatr. (2023) 35:189–204. doi: 10.1017/neu.2023.6, PMID: 36644883

[26] Rogowska M Thornton M Creese B Velayudhan L Aarsland D Ballard C . Implications of adverse outcomes associated with antipsychotics in older patients with dementia: a 2011–2022 update. Drugs Aging. (2023) 40:21–32. doi: 10.1007/s40266-022-00992-5, PMID: 36513918 PMC9747539

[27] Caspersen CJ Powell KE Christenson GM. Physical activity, exercise, and physical fitness: definitions and distinctions for health-related research. Public Health Rep. (1985) 100:126–31. PMID: 3920711 PMC1424733

[28] Sacheli MA Neva JL Lakhani B Murray DK Vafai N Shahinfard E . Exercise increases caudate dopamine release and ventral striatal activation in Parkinson’s disease. Mov Disord. (2019) 34:1891–900. doi: 10.1002/mds.27865, PMID: 31584222

[29] Tanaka S Yamagami T Yamaguchi H. Effects of a group-based physical and cognitive intervention on social activity and quality of life for elderly people with dementia in a geriatric health service facility: a quasi-randomised controlled trial. Psychogeriatrics. (2021) 21:71–9. doi: 10.1111/psyg.12627, PMID: 33140499

[30] Telenius EW Engedal K Bergland A. Effect of a high-intensity exercise program on physical function and mental health in nursing home residents with dementia: an assessor blinded randomized controlled trial. PLoS One. (2015) 10:e0126102. doi: 10.1371/journal.pone.0126102, PMID: 25974049 PMC4431827

[31] Vitale C Rosa R Agosti V Siciliano M Barra G Maggi G . Effects of Biodanza(®) SRT on motor, cognitive, and behavioral symptoms in patients with Parkinson's disease: a randomized controlled study. J Pers Med. (2024) 14:588. doi: 10.3390/jpm14060588, PMID: 38929809 PMC11204495

[32] Ferrero-Arias J Goñi-Imízcoz M González-Bernal J Lara-Ortega F da Silva-González Á Díez-Lopez M. The efficacy of nonpharmacological treatment for dementia-related apathy. Alzheimer Dis Assoc Disord. (2011) 25:213–9. doi: 10.1097/WAD.0b013e3182087dbc, PMID: 21346517

[33] Maher S Donlon E Mullane G Walsh R Lynch T Fearon C. Treatment of apathy in Parkinson's disease and implications for underlying pathophysiology. J Clin Med. (2024) 13:13. doi: 10.3390/jcm13082216, PMID: 38673489 PMC11051068

[34] Sajatovic M Ridgel A Walter E Tatsuoka C Colon-Zimmermann K Ramsey R . A randomized trial of individual versus group-format exercise and self-management in individuals with Parkinson's disease and comorbid depression. Patient Prefer Adherence. (2017) 11:965–73. doi: 10.2147/ppa.S135551, PMID: 28579759 PMC5449131

[35] Oba H Kobayashi R Kawakatsu S Suzuki K Otani K Ihara K. Non-pharmacological approaches to apathy and depression: a scoping review of mild cognitive impairment and dementia. Front Psychol. (2022) 13:815913. doi: 10.3389/fpsyg.2022.815913, PMID: 35250746 PMC8888661

[36] Prange S Klinger H Laurencin C Danaila T Thobois S. Depression in patients with Parkinson's disease: current understanding of its neurobiology and implications for treatment. Drugs Aging. (2022) 39:417–39. doi: 10.1007/s40266-022-00942-1, PMID: 35705848 PMC9200562

[37] Lautenschlager NT Cox KL Flicker L Foster JK van Bockxmeer FM Xiao J . Effect of physical activity on cognitive function in older adults at risk for Alzheimer disease: a randomized trial. JAMA. (2008) 300:1027–37. doi: 10.1001/jama.300.9.1027, PMID: 18768414

[38] Cugusi L Solla P Serpe R Carzedda T Piras L Oggianu M . Effects of a Nordic walking program on motor and non-motor symptoms, functional performance and body composition in patients with Parkinson's disease. NeuroRehabilitation. (2015) 37:245–54. doi: 10.3233/nre-151257, PMID: 26484516

[39] Alty J Farrow M Lawler K. Exercise and dementia prevention. Pract Neurol. (2020) 20:234–40. doi: 10.1136/practneurol-2019-002335, PMID: 31964800

[40] Felez-Nobrega M Haro JM Erickson KI Koyanagi A. Physical activity is associated with fewer subjective cognitive complaints in 47 low- and middle-income countries. J Am Med Dir Assoc. (2020) 21:1423–1429.e2. doi: 10.1016/j.jamda.2020.02.014, PMID: 32253161

[41] Soong RY Low CE Ong V Sim I Lee C Lee F . Exercise interventions for depression, anxiety, and quality of life in older adults with cancer: a systematic review and meta-analysis. JAMA Netw Open. (2025) 8:e2457859. doi: 10.1001/jamanetworkopen.2024.57859, PMID: 39903465 PMC11795328

[42] Tang L Zhang L Liu Y Li Y Yang L Zou M . Optimal dose and type of exercise to improve depressive symptoms in older adults: a systematic review and network meta-analysis. BMC Geriatr. (2024) 24:505. doi: 10.1186/s12877-024-05118-7, PMID: 38849780 PMC11157862

[43] Deeks JJ Higgins JPT Altman DG Cochrane Statistical Methods Group. Analysing data and undertaking meta-analyses. In: JPT Higgins J Thomas J Chandler M Cumpston T Li MJ Page, et al. editors. Cochrane handbook for systematic reviews of interventions, 2nd Edn. Chichester (UK): John Wiley & Sons. (2019) 241–84.

[44] Higgins JP Thompson SG Deeks JJ Altman DG. Measuring inconsistency in meta-analyses. BMJ. (2003) 327:557–60. doi: 10.1136/bmj.327.7414.557, PMID: 12958120 PMC192859

[45] D'Cunha NM Isbel ST Frost J Fearon A McKune AJ Naumovski N . Effects of a virtual group cycling experience on people living with dementia: a mixed method pilot study. Dementia (London). (2021) 20:1518–35. doi: 10.1177/1471301220951328, PMID: 32820955

[46] Hashimoto H Takabatake S Miyaguchi H Nakanishi H Naitou Y. Effects of dance on motor functions, cognitive functions, and mental symptoms of Parkinson's disease: a quasi-randomized pilot trial. Complement Ther Med. (2015) 23:210–9. doi: 10.1016/j.ctim.2015.01.010, PMID: 25847558

[47] Schaible F Maier F Buchwitz TM Schwartz F Hoock M Schönau E . Effects of Lee Silverman voice treatment BIG and conventional physiotherapy on non-motor and motor symptoms in Parkinson’s disease: a randomized controlled study comparing three exercise models. Ther Adv Neurol Disord. (2021) 14:1756286420986744. doi: 10.1177/1756286420986744, PMID: 33680093 PMC7897809

[48] Solla P Cugusi L Bertoli M Cereatti A Della Croce U Pani D . Sardinian folk dance for individuals with Parkinson’s disease: a randomized controlled pilot trial. J Altern Complement Med. (2019) 25:305–16. doi: 10.1089/acm.2018.0413, PMID: 30624952

[49] Lieberman A. Are dementia and depression in Parkinson’s disease related? J Neurol Sci. (2006) 248:138–42. doi: 10.1016/j.jns.2006.05.022, PMID: 16814323

[50] Dujardin K Sockeel P Devos D Delliaux M Krystkowiak P Destée A . Characteristics of apathy in Parkinson’s disease. Mov Disord. (2007) 22:778–84. doi: 10.1002/mds.21316, PMID: 17290451

[51] Pagonabarraga J Kulisevsky J Strafella AP Krack P. Apathy in Parkinson’s disease: clinical features, neural substrates, diagnosis, and treatment. Lancet Neurol. (2015) 14:518–31. doi: 10.1016/s1474-4422(15)00019-8, PMID: 25895932

[52] Fitts W Weintraub D Massimo L Chahine L Chen-Plotkin A Duda JE . Caregiver report of apathy predicts dementia in Parkinson’s disease. Parkinsonism Relat Disord. (2015) 21:992–5. doi: 10.1016/j.parkreldis.2015.06.009, PMID: 26117435 PMC4509809

[53] Tumati S Herrmann N Perin J Rosenberg PB Lerner AJ Mintzer J . Measuring clinically relevant change in apathy symptoms in ADMET and ADMET 2. Int Psychogeriatr. (2024) 36:1232–44. doi: 10.1017/s1041610224000711, PMID: 39297292 PMC11695175

[54] Wang Y Parpia S Couban R Wang Q Armijo-Olivo S Bassler D . Compelling evidence from meta-epidemiological studies demonstrates overestimation of effects in randomized trials that fail to optimize randomization and blind patients and outcome assessors. J Clin Epidemiol. (2024) 165:111211. doi: 10.1016/j.jclinepi.2023.11.001, PMID: 37939743

[55] Chong TT Husain M. The role of dopamine in the pathophysiology and treatment of apathy. Prog Brain Res. (2016) 229:389–426. doi: 10.1016/bs.pbr.2016.05.007 27926449

[56] Hamer M Chida Y. Physical activity and risk of neurodegenerative disease: a systematic review of prospective evidence. Psychol Med. (2009) 39:3–11. doi: 10.1017/s0033291708003681, PMID: 18570697

[57] Silva MVF Loures CMG Alves LCV de Souza LC Borges KBG Carvalho MG. Alzheimer’s disease: risk factors and potentially protective measures. J Biomed Sci. (2019) 26:33. doi: 10.1186/s12929-019-0524-y, PMID: 31072403 PMC6507104

[58] Del Prete M Spaccavento S Craca A Fiore P Angelelli P. Neuropsychiatric symptoms and the APOE genotype in Alzheimer’s disease. Neurol Sci. (2009) 30:367–73. doi: 10.1007/s10072-009-0116-9, PMID: 19590821

[59] Snowden JS Rollinson S Thompson JC Harris JM Stopford CL Richardson AMT . Distinct clinical and pathological characteristics of frontotemporal dementia associated with C9ORF72 mutations. Brain. (2012) 135:693–708. doi: 10.1093/brain/awr355, PMID: 22300873 PMC3286329

[60] Le Heron C Holroyd CB Salamone J Husain M. Brain mechanisms underlying apathy. J Neurol Neurosurg Psychiatry. (2019) 90:302–12. doi: 10.1136/jnnp-2018-318265, PMID: 30366958 PMC6518466

[61] Ballard C Fossey J Chithramohan R Howard R Burns A Thompson P . Quality of care in private sector and NHS facilities for people with dementia: cross sectional survey. BMJ. (2001) 323:426–7. doi: 10.1136/bmj.323.7310.426, PMID: 11520838 PMC37551

[62] Jao YL Loken E MacAndrew M Van Haitsma K Kolanowski A. Association between social interaction and affect in nursing home residents with dementia. Aging Ment Health. (2018) 22:778–83. doi: 10.1080/13607863.2017.1304526, PMID: 28332405

[63] Mok VCT Cai Y Markus HS. Vascular cognitive impairment and dementia: mechanisms, treatment, and future directions. Int J Stroke. (2024) 19:838–56. doi: 10.1177/17474930241279888, PMID: 39283037 PMC11490097

[64] Fisher BE Wu AD Salem GJ Song J Lin CH(J) Yip J . The effect of exercise training in improving motor performance and corticomotor excitability in people with early Parkinson’s disease. Arch Phys Med Rehabil. (2008) 89:1221–9. doi: 10.1016/j.apmr.2008.01.013, PMID: 18534554 PMC2989816

[65] Cassilhas RC Viana VA Grassmann V Santos RT Santos RF Tufik SERGIO . The impact of resistance exercise on the cognitive function of the elderly. Med Sci Sports Exerc. (2007) 39:1401–7. doi: 10.1249/mss.0b013e318060111f, PMID: 17762374

[66] Erickson KI Voss MW Prakash RS Basak C Szabo A Chaddock L . Exercise training increases size of hippocampus and improves memory. Proc Natl Acad Sci. (2011) 108:3017–22. doi: 10.1073/pnas.1015950108, PMID: 21282661 PMC3041121

[67] Lightman SL Birnie MT Conway-Campbell BL. Dynamics of ACTH and cortisol secretion and implications for disease. Endocr Rev. (2020) 41:41. doi: 10.1210/endrev/bnaa002, PMID: 32060528 PMC7240781

[68] Matmati J Verny C Allain P. Apathy and Huntington’s disease: a literature review based on PRISMA. J Neuropsychiatry Clin Neurosci. (2022) 34:100–12. doi: 10.1176/appi.neuropsych.21060154, PMID: 34961332

[69] Pagonabarraga J Kulisevsky J. Apathy in Parkinson's disease. Int Rev Neurobiol. (2017) 133:657–78. doi: 10.1016/bs.irn.2017.05.025, PMID: 28802937

[70] Wu D Zhang B Chang Y Huang S. Apathy associated with Alzheimer's disease. Curr Alzheimer Res. (2024) 21:527–37. doi: 10.2174/0115672050350970241216072400, PMID: 39716787 PMC12079319

